# Successful Resuscitation of a Patient with Life-Threatening Metabolic Acidosis by Hemodialysis: A Case of Ethylene Glycol Intoxication

**DOI:** 10.1155/2017/9529028

**Published:** 2017-07-25

**Authors:** Ikuyo Narita, Michiko Shimada, Norio Nakamura, Reiichi Murakami, Takeshi Fujita, Wakako Fukuda, Hirofumi Tomita

**Affiliations:** ^1^Department of Cardiology and Nephrology, Hirosaki University Graduate School of Medicine, Hirosaki, Japan; ^2^Community Medicine, Hirosaki University Graduate School of Medicine, Hirosaki, Japan; ^3^Murakami Shinmachi Hospital, Aomori, Japan

## Abstract

**Background:**

Ethylene glycol intoxication causes severe metabolic acidosis and acute kidney injury. Fomepizole has become available as its antidote. Nevertheless, a prompt diagnosis is not easy because patients are often unconscious. Here we present a case of ethylene glycol intoxication who successfully recovered with prompt hemodialysis.

**Case Presentation:**

A 52-year-old Japanese male was admitted to a local hospital due to suspected food poisoning. The patient presented with nausea and vomiting, but his condition rapidly deteriorated, with worsening conscious level, respiratory distress requiring mechanical ventilation, hypotension, and severe acute kidney injury. He was transferred to the university hospital; hemodialysis was initiated because of hyperkalemia and severe metabolic acidosis. On recovering consciousness, he admitted having ingested antifreeze solution. Thirty-seven days after admission, the patient was discharged without requiring HD.

**Conclusions:**

We reported a case of ethylene glycol intoxication who presented with a life-threatening metabolic acidosis. In a state of severe circulatory shock requiring catecholamines, hemodialysis should be avoided, and continuous hemodiafiltration may be a preferred approach. However, one should be aware of the possibility of intoxication by unknown causes, and hemodialysis could be life-saving with its superior ability to remove toxic materials in such cases.

## 1. Introduction

**Figure 1:**
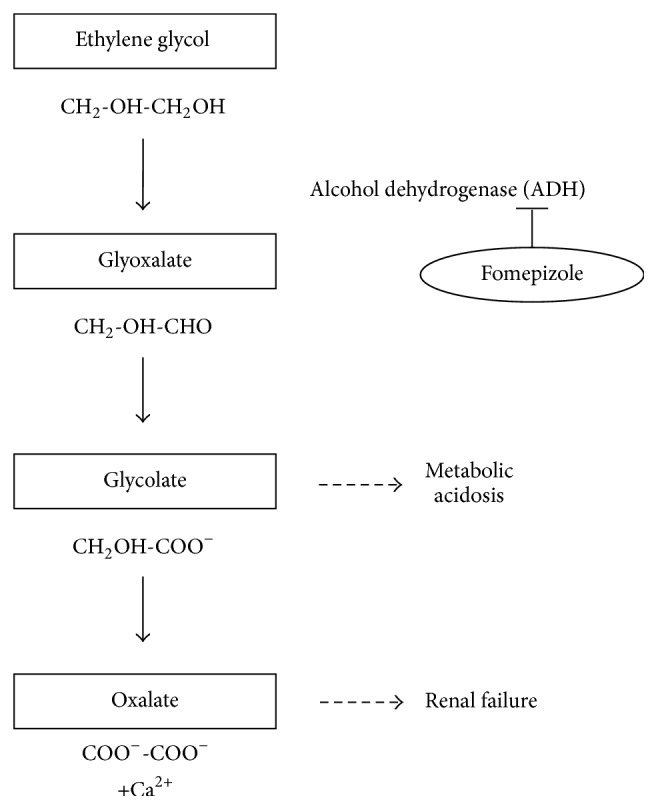
Metabolism of ethylene glycol and its mechanism of toxicity. Fomepizole acts as inhibitor of alcohol dehydrogenase and, therefore, prevents the formation of acidic ethylene glycol metabolites.

Ethylene glycol is used in commercially available products such as antifreeze solution, windshield, or cold-pack and is readily accessible in the household. It is odorless and has a sweet taste; therefore, accidental or suicidal ingestion may occur. Ethylene glycol poisoning is relatively uncommon, although it is an important cause of intoxication. In 2013, the American Association of Poison Control Centers Toxic Exposure Surveillance System reported 699 cases of ethylene glycol poisoning, resulting in 7 deaths, and this number should have been underestimated [1]. Ethylene glycol toxicity is related to the production of toxic metabolites by alcohol dehydrogenase (ADH) and aldehyde dehydrogenase that result in high anion gap metabolic acidosis (Figure 1). Calcium oxalate is produced from oxalates, and its precipitation in the renal tubules is one of the suggested causes of acute kidney injury. Therefore, the presence of calcium oxalate crystals in urinary sediment is an important clue for the diagnosis of ethylene glycol poisoning. Fomepizole, an inhibitor of ADH, is an antidote that prevents the production of toxic metabolites from ethylene glycol. Previously, ethanol infusion was widely used to inhibit ADH; however, the American Academy of Toxicology recommends treatment with fomepizole rather than ethanol, if available [2]. Fomepizole is used as an antidote for methanol intoxication as well. In the US, fomepizole was approved for the treatment of ethylene glycol intoxication in 1997, and in Japan, it was approved in 2015. However, it is not always easy to diagnose ethylene glycol intoxication because patients are often unconscious or small children [3] or have a cognitive impairment [4], and the use of fomepizole is limited to cases in which there is some clue of ethylene glycol ingestion.

Here we present a case of a life-threatening metabolic acidosis and acute kidney injury due to ethylene glycol intoxication who successfully recovered with prompt hemodialysis.

## 2. Case Presentation

A 52-year-old Japanese male was admitted to the gastroenterology department of a local hospital with suspected food poisoning. He presented with nausea and vomiting and had to be transported via an ambulance because he was unable to walk. On admission, he was awake, and his blood pressure and renal function were normal. During the next 24 hours (h), his condition rapidly deteriorated, with worsening conscious level, respiratory distress requiring mechanical ventilation, decreased blood pressure, and severe acute kidney injury. Thus, he was transferred to the university hospital. Laboratory data in the first hospital were shown in Table 1. He developed anuria and his initial arterial blood gas analysis showed a high anion gap metabolic acidosis, with the following results: pH, 6.92; pCO2, 20.6 mmHg; pO2, 538 mmHg; bicarbonate, 4.1 mmol/L; base excess, −30.3 mmol/L; anion gap, 15.9 mmol/L; and lactate, 21 mmol/L, under mechanical ventilation. Physical examination showed that his body temperature was 38.9°C, blood pressure was 80/50 mmHg, and pulse rate was regular at 106 beats/min. No remarkable findings were observed in his chest and abdomen, but his legs showed cyanosis. Laboratory tests revealed an increased white blood cell count of 36800/*μ*L. Serum creatinine (Cr) levels were elevated at 3.27 mg/dL, and potassium levels were 7.0 mEq/L. In order to improve hyperkalemia and severe metabolic acidosis, hemodialysis (HD) was initiated within 1 h on arrival. HD was performed under norepinephrine infusion for 4 h and followed by continuous hemodiafiltration (CHDF). Metabolic acidosis was dramatically improved, and on day 2 of admission his respiratory condition improved and he was extubated. On recovering consciousness, he said that he ingested antifreeze solution 6 to 12 h before the admission to the first hospital because it was sweet. He denied having committed suicide. We also referred him to the psychiatrist, and mental illness was denied. He denied simultaneous ethanol ingestion. Thus we suspected that his illness was caused by ethylene glycol intoxication. Anuric phase was persistent for the next 7 days; therefore, he had been on CHDF during his stay in the ICU. On day 9, he was shifted from ICU to the general ward, and intermittent HD was initiated three times a week for 4 h. HD was discontinued on day 26 of admission because his Cr levels had decreased to 2.3 mg/dL and urine volume had increased. The patient was discharged on day 37 of admission. Later, serum ethylene glycol level on admission to our hospital turned out to be 15 mg/dL. In 3 months, his Cr levels returned to normal.

## 3. Discussion

In this case, the patient was initially suspected as having food poisoning, but none of his family members who had the same meal had any digestive symptoms. Then, his condition rapidly deteriorated, and his doctor made an emergency call to a nephrologist in the university hospital, early in the morning. Food poisoning and acute kidney injury suggested the possibility of hemolytic uremic syndrome, but hemolytic anemia or low platelet count was not observed. At this time, the patient was transferred via an ambulance, and due to his life-threatening conditions, he was directly admitted to the ICU. His blood gas analysis revealed a high anion gap and severe metabolic acidosis, and his serum potassium level was 7.0 mEq/L. At this point, neither we nor his family was aware of the possibility that he had ingested ethylene glycol. On recovering consciousness, he admitted having ingested antifreeze solution because it was sweet. Usually, hemodialysis is avoided in cases with deteriorating vital signs with severe hypotension [5], and continuous hemodiafiltration may be preferred. However, fortunately, in this case, hemodialysis was selected because of patient's high potassium levels, and it was more efficient in removing ethylene glycol and its toxic metabolites.

It has been reported that clinically evident toxicity is usually seen with serum ethylene glycol levels of >20 mg/dL. A dose of >30 mg/dL is potentially lethal and that of 1.4 mL/kg is assumed to be lethal [6]. The patient stated that he ingested only a cap of antifreeze, and his ethylene glycol level (15 mg/dL) was below the toxic dose on arrival to our hospital, which was 48 to 72 h since ingestion. Considering that half-life of ethylene glycol is 3 to 9 h, the blood level at the time of admission to the first hospital should have been higher, possibly lethal level. It is possible that he did not remember the exact ingested amount. However, this case suggests that a relatively small amount of ethylene glycol can cause a life-threatening intoxication. At most facilities, the analysis of serum ethylene glycol levels requires several days. Thus, the general condition and the level of metabolic acidosis should be preferentially considered for therapeutic decisions. It has been suggested that hemodialysis is required despite the use of fomepizole in the event of metabolic acidosis with pH < 7.25, acute kidney injury or electrolyte imbalances that do not respond to conventional therapy, and deteriorating vital signs [5].

Previous case reports showed that the initial assessment took several hours, and 4 h after admission, either fomepizole [3] or hemodialysis [7] was initiated, and both cases were survived. In this case, we initiated hemodialysis within 1 h on arrival, and we assume that the prompt initiation of hemodialysis was an important factor in his survival.

## 4. Conclusions

We reported a case of ethylene glycol poisoning who presented with a life-threatening metabolic acidosis. In a state of severe circulatory shock requiring catecholamines, hemodialysis should be avoided, and continuous hemodiafiltration may be preferred. However, one should be aware of the possibility of intoxication by unknown causes, and hemodialysis can be life-saving with its superior ability to remove toxic materials.

## Figures and Tables

**Table 1:** Laboratory data in the first hospital.

Parameters	Day −1		Day 1	
	1 am	4 pm	9 pm	4 am
RBC (× 10^4^/*μ*L)	544	639	590	544
Hb (g/dL)	16.7	17	19.6	16.6
WBC (/*μ*L)	8,570	22,350	34,770	87,860
Plt (× 10^4^/*μ*L)	25.3	30.3	34.3	34.1
TP (g/dl)	7.8	10.3	9.0	7.8
Alb (g/dl)	5.1	6.7	5.8	5.0
LDH (IU/L)	176	347	254	386
BUN (mg/dl)	13.9	11.8	17.2	29.8
Cr (mg/dl)	0.69	0.94	1.38	3.1
eGFR (mL/min/1.73 m^2^)	91.2	65.1	42.7	17.6
Na (mEq/L)	142	144	141	145
K (mEq/L)	5.1	6.2	6.0	7.0
Cl (mEq/L)	107	117	115	115
Ca (mg/dl)	10.3	11.3	10.4	9.4
CRP (mg/dl)	<0.3	<0.3	<0.3	0.5
Arterial pH		7.07	7.0	6.8
PaO_2_ (mmHg)		138	209	211
PaCO_2_ (mmHg)		10	16	21
Bicarbonate (mmol/L)		2.8	7.8	6.2
Base excess (mmol/L)		−24	−25	−28

## Abbreviations

ADH: Alcohol dehydrogenase
Cr: Creatinine
HD: Hemodialysis
CHDF: Continuous hemodiafiltration
ICU: Intensive care unit.
